# Balancing Fidelity and Adaptation: Action‐Oriented Research Towards Implementing a Nutrition Education Program Among Adolescents

**DOI:** 10.1111/josh.70009

**Published:** 2025-05-04

**Authors:** Marion D. Driessen‐Willems, Nina H. M. Bartelink, Kathelijne M. H. H. Bessems, Stef K. Kremers, Patricia van Assema

**Affiliations:** ^1^ Department of Health Promotion Research Institute of Nutrition and Translational Research in Metabolism (NUTRIM), Maastricht University Maastricht the Netherlands

**Keywords:** action‐oriented research, adolescents, implementation fidelity and adaptation, nutrition education

## Abstract

**Background:**

Implementation of school‐based health promotion programs requires contextual fit. To strengthen the nutrition education program “Krachtvoer” (ENG: “Power Food”) and learn general lessons about contextual fit, this study examined how the program, the context, and program‐context interactions affected teachers' balancing between implementation fidelity and adaptation.

**Methods:**

As part of a co‐creation process with continuous micro‐process cycles of implementing, measuring, evaluating, and adapting the program, action‐oriented research was conducted during the pilot implementation of program modules by 25 teachers in 32 classes with 635 students. Using observations and interviews, data were collected about indicators of the implementation process, technology, layout, and content aspects of the program, inner and broader school contextual factors including teacher, student, and school characteristics, and interactions between program‐ and context‐related aspects that influence the implementation process.

**Results:**

Even small mismatches between the program and the context affected the implementation process. Differences in the technological savviness of teachers and students, “adaptive management” skills to respond to changing circumstances of teachers, and the maturity and attention span of students were among the many contextual differences in and between schools.

**Implication for School‐Based Health Promotion:**

Sustainability of health promotion programs fitting the context requires continuous and co‐creating efforts from all stakeholders.

**Conclusions:**

Action‐oriented research with micro‐process cycles proved appropriate for strengthening the program. However, further research is needed on capacity building among program implementers in balancing fidelity and adaptation.

## 1 Introduction

Evidence‐based school health promotion (HP) programs have the potential to promote students' health behavior [1, 2]. However, it often remains a challenge for the implementers of these programs, such as teachers, to implement them in their daily practice [3]. As a result, implementation is often suboptimal and the potential public health impact is not achieved [4, 5]. Schools can be seen as complex adaptive systems with a dynamic and complex nature, in which many actors and contextual factors are continuously changing and interacting [6, 7, 8]. This means that any implementation outcomes as indicators of the implementation process in schools are influenced not only by the characteristics of the HP program itself, but also by characteristics related to its actors (e.g., teachers and students), the inner school setting (e.g., school size), and the broader school setting (e.g., education sector) [9, 10]. Furthermore, the connections and interactions between a school and other systems, such as home and neighborhood, as well as the strategies used to support the implementation process (e.g., teacher training, helpdesk, and funding), influence the way HP programs are implemented [11, 12, 13]. This implies that the optimal implementation of HP programs is likely to vary across schools and requires HP programs and their implementation strategies to be flexible to realize a contextual fit with the school [14, 15].

Research towards implementation outcomes typically focuses on fidelity and thereby includes the primary indicators of dose (i.e., the amount of exposure to the intervention), adherence (i.e., implementation according to theoretical intervention principles), quality of delivery (i.e., how well the intervention is delivered), participant responsiveness (i.e., engagement with the intervention), and program differentiation (i.e., the implementation of essential interventions components) [16, 17, 18]. HP program developers often expect high implementation fidelity, operationalized as the degree to which an intervention was implemented as intended by the developers [16, 17, 18], to positively impact its intended intervention outcomes [19]. Rather, in practice, implementation is often characterized by adaptation: the implementer makes implementation decisions to adapt the program to the context and always creates an alternative implementation solution [19, 20]. Adaptation has been acknowledged as favorable, for example, in case an implementer modifies the HP program to meet the needs, interests, and opportunities of the context in which the intervention is implemented. However, adaptation can also be unfavorable if it compromises the function of the program, represented by the core characteristics and components involved in the behavior change process [21]. It is therefore important that already from the start of the development process, both program developers and implementers consider how to achieve a balance between fidelity and adaptation, guided by the context in which an HP program is implemented [6, 19, 22]. This requires that HP programs are developed to be sensitive and adaptable to different implementation contexts. Co‐creation, a dynamic process between researchers, implementers, and the target population, has been proposed as a proper strategy to generate new knowledge on how to make HP programs sensitive to their implementation context through honest, democratic, and meaningful engagement [23, 24]. Action‐oriented research, that is, research designed and conducted in close collaboration with the research population and other relevant societal stakeholders, can support the co‐creation approach by embracing and integrating “real world” dynamics [25].

This paper reports on action‐oriented research conducted as part of a co‐creation process of strengthening the Dutch classroom nutrition education program Krachtvoer (ENG: Power Food) for 12‐ to 14‐year‐old students in prevocational schools. The main themes are fruits, drinks, and healthy snacks. The 13 modules consist of many active and varied digital and nondigital didactical working methods, such as worksheet assignments, food preparation and tasting, and interactive games. The Krachtvoer.nu website serves as a broad support program for teachers with access to the teaching materials, including teachers' manuals, worksheets, and pre‐structured Digi‐board presentations with links to, for example, short videos and instructions for assignments (Box 1).

BOX 1 **Krachtvoer Intervention.**

Krachtvoer is a classroom educational intervention taught by teachers to first and second graders (aged 12–14 years) of prevocational schools, which offer education at four educational levels. The intervention's goal is to help students make lasting healthy lifestyle choices. The main themes are fruits, drinks, and healthy snacks. The intervention consists of 13 modules.

**Intervention History and Theoretical Backbone**

Krachtvoer has existed since 2002. An updated version of Krachtvoer was worked on in 2017 and has been available at www.krachtvoer.nu since July 2019. Krachtvoer has been systematically developed and adapted over time based on available theoretical and empirical knowledge and results from effect and process evaluation studies of Krachtvoer and has a phased approach [26, 27, 28]. The successive modules go through the steps necessary to achieve lasting behavioral change: from attention and enthusiasm for the subject of healthy eating and being aware of one's own food choices, via identifying and improving the behavioral determinants that may be a barrier to change for the target group itself, to formulating personal goals, to making, regularly evaluating and adjusting action plans. The intervention has been accredited by the National Institute for Public Health and the Environment based on its previous process and effect studies.

**Intervention Description**

The intervention consists of 13 modules of 30–45 min, including five fixed modules and eight modules from which the teacher can choose, responding to the needs of a class. Preferably, teachers implement the modules over at least eight lessons, and in eight consecutive weeks. In Krachtvoer, it is all about students being actively engaged in their own nutrition behavior. This is reflected in the many active and varied digital as well as nondigital didactic working methods, such as worksheet assignments, photo assignments, environmental scans, food preparation, tasting, quizzes, and interactive games. The website acts as a broad support program for use. Teachers register once on the website and then have access to the teaching materials, including teacher manuals, lesson plans with concise module overviews, worksheets for students, posters, ready‐to‐use and pre‐structured Digi‐board presentations with links to short videos, interactive games and quizzes, and instructions for assignments (see the example below). The website also includes a student webpage. In some modules, students visit the student webpage of Krachtvoer via their internet‐connected device (e.g., mobile phone or tablet) or school computer. The student webpage includes tips, informative videos, interactive games, and a computer‐tailored “planning buddy” that students use to make and evaluate their own action plans. The webpage also includes links to external relevant web pages, and tools such as the online quiz application “Kahoot.” Students are also asked to download the app “Kies ik Gezond?” (ENG: “Do I choose healthy?”) of the Dutch Nutrition Centre, on their own devices. In some modules, students are asked to use the camera on their own devices to complete homework assignments and gain insight into their own fruit, snack, and drink consumption. The website and all the teaching materials are designed in their own house style, including a logo, color choice, embellished with small drawings, and three characters representing the intervention themes with a name, a voice, and a slogan (Fruit: “Fruity,” Drinks: “Thirsty,” and snacks: “Snacko”).

**Additional Teacher Support**

Teachers find step‐by‐step support on the website to get started with Krachtvoer, and each module includes a range of teacher support options, such as substantive background information on nutrition or tips for interacting with students. Teachers can use the support options depending on their own needs and the specifics of their students. In addition, opportunities for intervention expansions are offered within the nutritional theme and to other lifestyle behaviors, which could fit well in some schools. Besides being encouraged and supported to adapt the choice of the modules to their own context, teachers are provided with alternative didactic working methods throughout the modules that might fit their context better. Nondigital alternatives are provided for lesson components that require students to use a device.

**Example Digi‐Board Presentation Module 8 the School Environment**

Sheet 1: Do you make your own lifestyle choices? Or are you influenced by your environment? Buttons to two short videos introducing this lesson topic.

Sheet 2: Two written examples of environmental influences to be used by the teachers to further introduce the lesson topic: a canteen that primarily offers unhealthy food and hidden stairs.

Sheet 3: Instructions for the assignment in which the students scan the school environment in small groups using the worksheet of the module, which includes a pre‐structured observation form.

Sheet 4: Questions to guide the plenary debriefing of the assignment.

**FIGURE 1:**
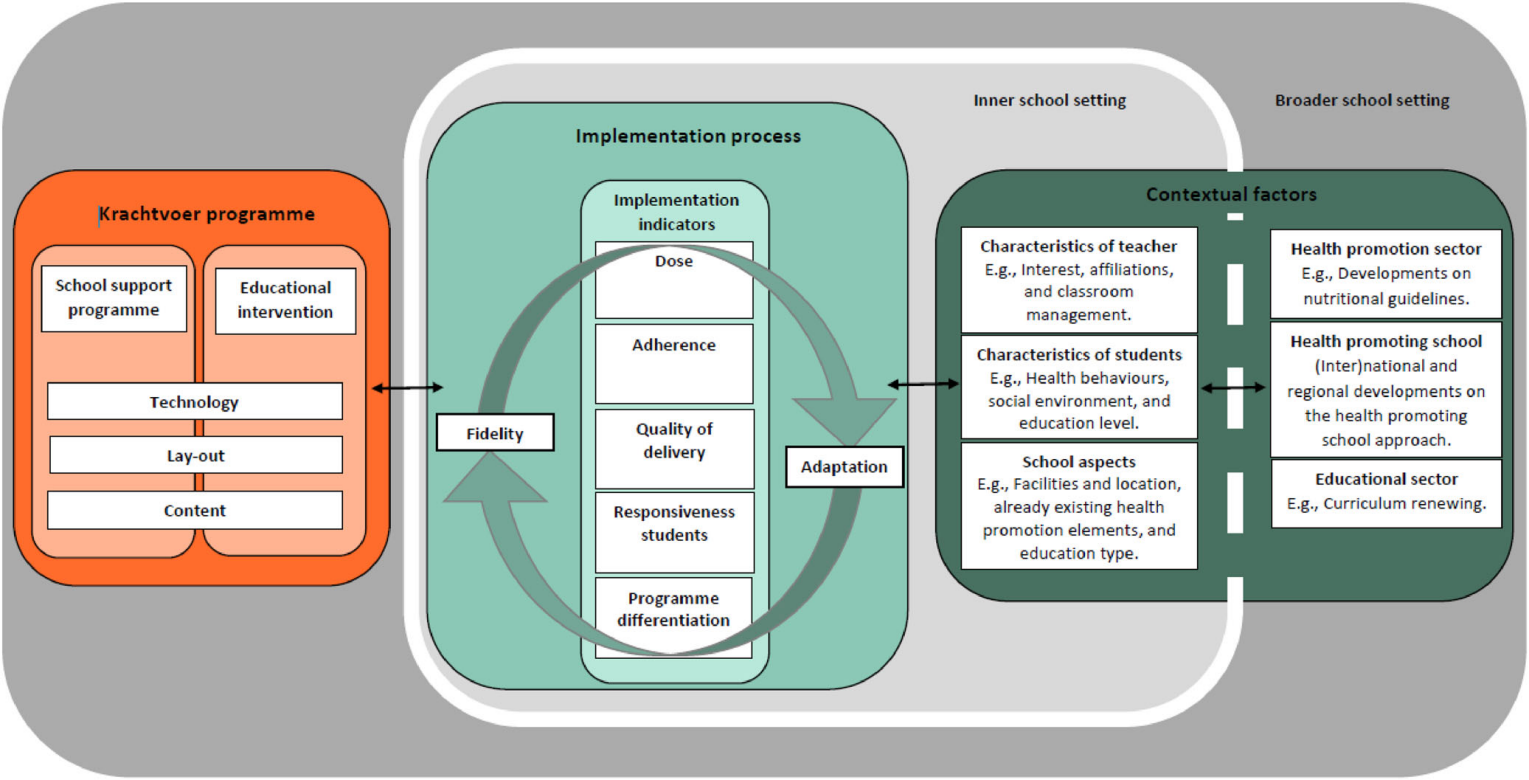
Conceptual framework of contextual fit [26].

The Conceptual Framework of Contextual Fit (Figure 1) guided this study [26]. The rationale of the framework is that the program's implementation process is determined by the technology, layout, and content aspects of the program, the continuously changing inner and broader school contexts, and the interaction between these program‐ and context‐related aspects. To strengthen the Krachtvoer program and to learn general lessons about contextual fit, this study aimed to generate insights into how teachers balanced between fidelity and adaptation during the actual implementation process of Krachtvoer by examining how the program, context, and program‐context interactions affected the implementation process.

## 2 Methods

### 2.1 Setting

The current study was part of the co‐creation approach to revise Krachtvoer that has started in 2017 [26]. The co‐creation is a joint initiative of HP experts from Maastricht University and professionals from the education organization Yuverta. Prevocational education locations in Yuverta act as primary collaboration partners in the co‐creation, enabling the direct connections with students, teachers, parents, and school support staff. At the national level, an intersectoral network of partner organizations supports the co‐creation process. A first co‐creation aim was to evolve the formerly paper version of Krachtvoer into an online accessible intervention that is accompanied by a broad user support program. In addition, the continuous process of co‐creation aims to strengthen Krachtvoer by developing it into an intervention that can be adapted to the ever‐changing implementation context and to the unique context in each school. The approach is driven by continuous micro‐process cycles of implementing, measuring, evaluating, and adapting the program. The current study took place in the second phase of the co‐creation process, after a draft online version was developed in the first phase.

### 2.2 Study Design

An action‐oriented research design was used with observations and different types of interviews. During the first 3 months of the school year 2018–2019, the draft version of the Krachtvoer program was implemented at four Yuverta school sites. The deputy principal and/or principal of the sites determined when and how (i.e., in which course, by which teachers and classes) Krachtvoer could be implemented. No strict research goals were set in terms of the number of lessons to be implemented. An agreement was made with the co‐creation schools that they would try to implement lessons whenever possible during the short study period. On their own initiative, a phased start was achieved, with two sites starting implementation at the beginning of the study period, seamlessly alternated by the two other sites. Consistent with the idea of the micro‐process cycles in the co‐creation approach, once a lesson had been implemented, the findings were fed back as quickly as possible to all those involved and discussed during the co‐creation meetings. Subsequently, necessary program adjustments were made. The next time a lesson was implemented, the latest version of the program was used, thus starting a new cycle. This allowed program changes made at the beginning of the study period to be re‐evaluated later in the study period.

### 2.3 Participants

Twenty‐five teachers participated in the study. Fifteen teachers were experienced teachers who had been teaching for more than 10 years. Nine teachers taught Biology or Personal care and implemented Krachtvoer modules within those classes. Sixteen teachers taught different subjects, but were also the mentor teacher of all students in a number of classes. They implemented the Krachtvoer lessons during scheduled mentor hours for two classes at the same time. In total, the participating teachers implemented Krachtvoer lessons in 32 classes with a total of 635 students, in 104 “sessions” accounting for 150 h. Ranging between a minimum of 4 modules to a maximum of 13 modules, most teachers were able to implement 8 modules.

### 2.4 Data Collection Methods

Multiple methods were used (Table 1), each assessing a specific part or all of the concepts from the underlying conceptual framework, that is, indicators of the implementation process and potential influential technology, layout, and content aspects of the program, factors in the inner and broader school contexts, and interactions between these program‐ and context‐related aspects.

**TABLE 1:** Data collection methods.

Survey	Prior to data collection at the school, the participating teachers were asked to fill out a short survey, sent to them by mail, about their age, years of teaching experience, and the subjects they teach. They were also asked to specify in how many and which classes they would implement Krachtvoer, that is, first or second graders, and the educational level of the students and the subject they teach in those classes.
Observations and student questioning	Most lessons were observed by the same two researchers and some by one of these two researchers, using semi‐structured observation forms addressing all main concepts of the study's conceptual framework. The researchers discussed their observation results directly afterward, and if necessary, consensus was sought to finalize the results. During the observed lesson, the teacher, at the request of the researcher(s), asked questions of the students to better interpret observations, for example, why they laughed during an assignment. These questions and answers were noted by the researchers on the observation form.
Semi‐structured interviews	After each observed lesson, short interviews of a few minutes took place during the teacher's break or before the next lesson began, by the same two researchers and some by one of these two researchers, using a list of questions based on the study's entire conceptual framework. For each lesson, the researcher(s) selected and prioritized the questions from the list that were most relevant given the course of the lesson. Notes were taken by the researcher(s) during the interview and completed into a report immediately after the interview. All interviews were recorded, but only listened back in the sporadic case of an incomplete report. Occasionally, teachers wanted to add something to their interview answers afterward and did so via e‐mail. This information was added to the reports.
Focus group interviews	Two focus groups were held with students from two schools midway through the study period. The students were asked to discuss the program characters, verify observation and interview results from the first half of the study period, and generate ideas for adapting the program characters. Both focus groups included eight students, lasted approximately 15 min, and took place in a classroom. Notes were taken by the main researcher during the interviews and completed into a report immediately after the interviews. The interviews were recorded and listened back to complete the report.
Plenary evaluation session	At the end of the study period, a one‐and‐a‐half‐hour plenary evaluation session took place on a teacher study day, to which all participating teachers from the four participating schools were invited. In this session, a discussion was initiated based on statements made by the researchers on those aspects of the program for which the other methods of data collection revealed multiple but sometimes opposing views of the teachers, for example, I find the learning methods too inactive, the program's website user‐friendly, the characters fit the target group, my students really learn from the Krachtvoer lessons. Twenty out of 25 teachers were present at the evaluation session. The session was recorded and transcribed verbatim.

### 2.5 Data Processing and Analysis

Descriptive statistics were used to process the survey data. The Nvivo program version 12 (QRD International Pty Ltd., Australia) was used to categorize and analyze the data in the reports. The coding of the data by the main researcher (M.D.) primarily aimed to order the data according to the concepts of the study's framework and the presumed interactions between the concepts. We analyzed the data from three different perspectives to gain insight into the many complex relationships between concepts in the conceptual framework. First, we focused on the identification of the characteristics of the program that promote or inhibit the implementation in the general prevocational school context. Second, we focused on the interactions of program characteristics with unique contextual characteristics and their influence on implementation. Third, we focused on program adaptations and whether the program sufficiently facilitates adaptation.

## 3 Results

### 3.1 Program Characteristics Related to Implementation of Fidelity Indicators in the General Prevocational School Context

Observations and interviews with teachers revealed that the ready‐made and pre‐structured learning materials were extremely important for program adherence. The Digi‐board sheets allowed teachers to go through the various program components in the correct order, which happened naturally. The videos, quizzes, and assignments that were launched through these sheets captured the content of these program components. The worksheets also ensured that the assignments were introduced by the teacher and executed by the students as intended (promoting adherence).

Students indicated that the Krachtvoer Digi‐board lessons and worksheets were more appealing than other educational materials. The design of the materials, including the colors, pictures, and logo, was generally well‐received by the target group. Students typically enjoyed coloring the small drawings in the materials. Teachers, students, and school support staff frequently expressed that they considered the design to be of a quality that one can expect these days. Many stated that this increased their willingness and enjoyment of working with the program (promoting adherence and responsiveness). Teachers did express the need, though, for black‐and‐white printable materials to keep costs low and to be able to have printed materials for all students (promoting dose).

According to both teachers and students, especially the many active learning methods in the program fit well with the target group. Many teachers said that the active methods were popular among students because they fit students' needs to work hands‐on and independently. Teachers stated that most of their prevocational students want to keep up the pace, but are also quickly saturated and bored. Teaching materials such as worksheets and the student webpage allowed students to be actively involved in their own learning and with control over their own work, which students often appreciated. The observations showed that especially the digital program components in simple language engaged students better in the program for a longer time (Table 2, Citation 1) (promoting responsiveness).

**TABLE 2:** Citations of teachers and students.

Number	Citation
1	[Students enter the classroom] “Oh, we are going to do Krachtvoer again. That is more fun than a normal lesson because we can use our phone.” (student)
2	“I experienced the study period as very educational. It gives a different perspective on the content of my biology teaching. And it gives insight into how our children today like to learn. That is a welcome addition of this project to my own way of teaching.” (teacher).
3	“But no one has to tell me what to eat.” (student) “These lessons are intended to familiarize you with what is healthy, and what is not so healthy. We don't want to force you to change your eating pattern; you remain responsible for that.” (teacher)
4	“Nice that different forms of work are used. With prevocational students the tension curve is short. By alternating the methods, it is easy for them to keep up. Nice to have a combination of listening and more active work forms.” (teacher)
5	“Different methods are necessary to keep the program interesting for the students. But in one lesson, the methods should not vary too much. Bringing the group together again and again takes a lot of energy, including from my students.” (teacher).
6	“It feels a little restless to me, so much difference and switching between methods. It's better not to keep going back to plenary, but to let them keep working on their own.” (teacher)
7	“I looked at all the modules and found these modules to be the most active, in which students can also work independently in groups. I deliberately did not choose modules with actual food products, because I find that difficult in this classroom. I prefer to do that in a cooking classroom.” (teacher)
8	“Because of budget cuts, many students from special education come to this regular education program but cannot handle it at all. In general, every student seems to have something.” (teacher)
9	“If you don't eat snacks, then you really don't have a life.” [laughing] (student).
10	“Due to this being a pilot, these lessons are not planned in my Biology course. So I am linking with my lessons on plants in year 1 and nutrition in year 2. But then we haven't finished the teaching material and we need to do that for the test. So I have to see how I will do that, because 8 lessons are too much for me. When we have had 4, we will see how far we are.” (teacher)
11	“Perhaps I am exaggerating too much for the average teacher. Of course, I have 20 years of experience with health education. It is not important to me that it has to fit into three quarters of an hour. Because even though I have not finished my lesson, I have achieved the lesson goal: being conscious of what you put in your mouth and why. Being confronted with the fact that things really need to be different. And then realizing it. The moment I achieve that with 80% of the students, I don't finish the lesson. That's what I have learnt from this program.” (teacher)
12	“I liked the lessons because I think the subject is fun and important. I really think this is one of those lessons that you can apply in your life. I think the lessons are well designed.” (teacher)

Most teachers initially expressed doubts that the characters were too childish for their students, decreasing their enthusiasm to work with the program (inhibiting the quality of delivery). Some teachers explained that they had started nonetheless, and after a while, the students did not seem to care too much about the characters. The observations showed that some other teachers avoided program components that featured the characters, for example, the videos (inhibiting dose), as they explained in interviews for fear of losing their grip on the students by offering material that was too childish. Most teachers were surprised that when students were asked for their opinions about the characters, most students were quite positive (promoting responsiveness). Often students indicated that the characters reminded them of characters in some movies. This made teachers more at ease in implementing these program components after all. Students especially appreciated the visual aspects of the characters and described them as cute, colorful, and funny. Some students, on the other hand, found them boring or even scary, or indeed childish (both promoting and inhibiting responsiveness). Appreciated aural aspects of the characters, including their funny names. The female voice of one of the characters was considered too childish, annoying, and unrealistic. Even after changing and retesting, it remained the lowest‐rated voice. Most students found the characters' slogans funny and repeated them or came up with a sequel that rhymed (both promoting and inhibiting responsiveness).

Most teachers appreciated the choice between multiple learning methods, including the option to choose between program components that do or do not require students to use a device. They explained that it allowed them to choose methods that fit with their own and students' technological skills and preferences, ensuring a smooth course of the lesson (promoting quality of delivery). At the beginning of the study period, several technological glitches occurred on the website and when using the Digi‐board presentations, which hindered teachers in preparation, but also caused serious problems during the implementation of digital program components. It reinforced unwanted reactions from students, which appealed to teachers' classroom management skills, which in turn negatively affected implementation (inhibiting dose). The technological problems could all be resolved quickly, and later observations showed that these issues hardly caused any problems in the later study period. Similarly, several but minor language problems in the lesson materials were observed, that is, formulations that led to confusion or misunderstanding by one or more teachers and/or students (inhibiting adherence). These were adjusted during the study period until no more problems were observed with a particular wording.

Several teachers who were observed or indicated to be less technically savvy reported that they found it difficult to get a good program overview. Therefore, a printable lesson plan with the outline of the module was developed for each module during the study period. Observations and interviews in a later stage of the study period revealed that the lesson plans were appreciated by the teachers and helped them to keep track during the module. The lesson plans also supported teachers with little nutrition knowledge, giving them more confidence in their ability to implement the module (promoting adherence and quality of delivery). Another interim program addition to support less tech‐savvy teachers was the inclusion of print screens of digital program materials, such as Digi‐board sheets and worksheets, in the teacher manuals (promoting adherence). This addition was also confirmed as an improvement in the interviews in a later stage of the study period.

Regarding the design of the website, teachers expressed the importance for the content to be brief but powerful and active, and needing a minimal number of clicks to navigate through the website. If the website design did not meet these aspects, observations showed that it could lead to skipping program components (inhibiting dose) or rushing teachers (inhibiting adherence). The micro‐process cycles allowed resolving these issues.

Observations and interviews indicated that teachers did not use the opportunities in the support program to improve their own knowledge and skills to broaden the program and embed it in a whole‐school approach. The reasons given were that they found it too much work in combination with learning to work with a new program and the high workload in their daily practice, but especially that it was optional in the context of the research study, so they did not feel obligated to use it (inhibiting dose and quality of delivery). Nevertheless, in the interviews, most teachers said that they appreciated the support provided, understood its usefulness, and would like to use it in the future. Firmly anchoring the program in the school curriculum was considered essential for the sustainable use of the program (promoting adherence).

The value of the co‐creation approach itself was confirmed by the teachers' explanations of their gains from participating in the study in terms of new insights into teaching methods (Citation 2).

### 3.2 Interactions of the Program With Unique Contextual Factors

The observations revealed that the quality of program delivery was better among teachers who demonstrated better didactic skills. They were better at engaging students, remained calm and listened to students, dealt more adequately with student resistance, or had an affinity for HP and the subject of nutrition (Citation 3). Observations also showed that teachers' prior knowledge of nutrition contributed to higher quality of delivery, as those teachers were able to ask in‐depth questions that improved information processing by students. In the plenary evaluation, teachers with little nutrition knowledge stated that they considered nutrition education risky for the target group. They were afraid of triggering eating disorders. In the interviews, some teachers stated that they were alert to higher‐weight students being uncomfortable with the topic and stressed the importance of inclusiveness and avoiding stigma. In this regard, some teachers explained the added value of the program being taught by a teacher who has expertise in the subject of nutrition. Teachers who wanted to use their own familiar learning methods for their own convenience or because they thought it was best for their students had poorer program adherence than teachers who were more flexible in implementing suggested methods.

The interviews revealed differences in teachers' perceptions about the use of the many varied learning methods and the extent to which this suited them and their students. Many teachers said that varying methods within a single lesson keep students actively engaged and focused, although some of those teachers said that the methods should not be varied too quickly (Citations 4 and 5). Some teachers want to alternate methods as little as possible (Citation 6). Observations showed that especially the plenary learning methods required a lot of interaction between the teacher and students, which required great effort on the part of teachers. Therefore, once students worked well independently, some teachers decided to give some extra time for such an assignment at the expense of the next assignment with a method that would again require effort from the teacher.

A limiting contextual factor in some schools in implementing active learning methods was the classroom. Traditional biology classrooms have fixed taps and workbenches, which create obstacles and hinder group work. Also, the classrooms equipped for giving cookery lessons were not always available during scheduled classes, as some schools had only one or two such classrooms (Citation 7).

Several teachers reported that although the students enjoyed program components that required the use of a device, it caused undesirable responses in some students, such as entering odd profile names or becoming overexcited. This behavior inhibited getting to the core content of the particular educational task. In some cases, this caused time pressure, which led to teachers' perception of low learning efficiency among students. As a result, teachers skipped or shortened program components. In addition, it was frequently reported and observed that one or more students had no or a non‐working mobile phone. Further, in the interviews, several teachers expressed that especially first‐year students did not always have the required technological skills to connect to the Wi‐Fi network and work with the provided apps. Observations revealed that in all schools, the Wi‐Fi connection in some classes was not sufficient to meet demand. Relatedly, teachers, both experienced and somewhat less experienced, repeatedly indicated that they did not feel proficient in switching between the various digital technologies the program entailed. Also, the sound and image settings of the computer and the Digi‐board were not always good. As a result, for example, videos were unintelligible or too loud, or the readability of the Digi‐board sheets was poor.

Implementation did not go equally well with every group of students. Observations showed that, in general, the lower education level classes participated better than higher education level classes. Yet, teachers explained, and observations confirmed, that for the lowest level students, working with the program is the most challenging. Teachers further indicated that students' personal problems and mismatched educational levels are barriers (Citation 8). Several observations were made of situations in which the existing social norm and group behavior in a class influenced the implementation of the program. Teachers confirmed this influence and the fact that students at this age are sensitive to social norms. Examples of observations include students who felt nutrition was too personal and sensitive a topic to discuss in a full class and did not actively participate. There were also several observations of students using quotes that reflected specific normative beliefs on healthy nutrition in that class (Citation 9). Furthermore, observations revealed, and teachers confirmed differences in maturity between first‐ and second‐year students. This appeared to particularly influence their opinion of the program's design. Compared to the positive reactions of the younger students, some second‐year students found the appearance, names, voices, and slogans of the program characters childish and therefore stated that they did not like working with the program. Finally, the quality of performance of the small group assignments depended on the composition of the small groups. The observations showed that it was less adequate in groups of more than five students, mixed boy and girl groups, and groups with dominant students.

Poor preparation of the teacher due to lack of time or unplanned absence (e.g., illness) or bad timing of the lessons causing restlessness in the classroom was observed and reported as reasons for some teachers to rush through program components.

### 3.3 Program Adaptations

Most teachers adapted program components to best suit their own preferences and skills (e.g., digital literacy) and those of their students. This often occurred in line with suggestions in the program manual. For example, as the program suggested, teachers chose a more difficult or easier quiz, depending on the educational level of their students and the time available. In general, teachers adapted the implementation of the program to the time available (Citation 10).

The interviews and observations revealed that adaptations occurred when the teacher noticed the need for adaptation, felt the space for adaptation, and when the adaptation was consistent with the teacher's beliefs. Also, the interviews showed that the teachers with more years of teaching experience drew on experience and steered the lesson towards achieving the lesson goal and therefore adherence, while less experienced teachers tried to complete all lesson components (Citation 11).

In a few cases, observations showed that adaptation was not carried out but would have been necessary. This was caused by lack of experience with the program, being overburdened by the students, experiencing a high workload, having no affinity for the topic of nutrition, and/or having little motivation to implement the program due to the top‐down decision to implement the program.

Teachers interpreted the adaptation suggestions in the program as encouragement to adapt the lessons to their own students in their own way. Overall, teachers often showed resourcefulness in adapting the program to situations so that they could continue teaching, from their motivation to implement the lessons (Citation 12).

Observations showed that some adaptations were successful and favorable; for example, when a Kahoot quiz was not working properly, the teacher asked students to raise their hands to give the correct answer, and that worked fine as well. Or if teachers enriched the program on their own initiative by showing documentaries or bringing related news feeds to the students. Yet sometimes adaptations were less successful and unfavorable and led to students not wanting to participate actively, for example, when a teacher showed an English‐speaking documentary that was not understood by the Dutch students. Often, these unfavorable adaptations have their source in bypassing technological program components due to program malfunction or teachers not feeling competent to work with the provided (digital) implementation variant.

The success of the adaptations also depended on students' expectations. In one school, for example, the program was implemented in a project course for which students expected to entail mainly practical work. Some Krachtvoer lessons disappointed them because they found them boring and relatively theoretical. When the program was implemented in the biology course, students were generally more enthusiastic because the Krachtvoer lessons were more practical than most Biology lessons.

## 4 Discussion

Our study examined how the program, context, and program‐context interactions influenced the implementation of the Krachtvoer program. The aim was to strengthen the Krachtvoer program, but also to learn general lessons about the contextual fit of school‐based HP programs and balancing between program fidelity and adaptation.

### 4.1 Key Findings

As for program characteristics, pre‐structured materials, such as Digi‐board sheets, had an important role in promoting program adherence. Other applications of such materials, such as lesson plans, have also been tried in other programs [29, 30, 31, 32, 33]. Despite the structure provided, these materials proved to go well with stimulating program adaptation to one's unique context.

The study supports previous findings about the importance of appealing learning materials for program implementation [30, 32, 33], where this study found that not only is it important that students like the materials, but also that teachers' expectations of students' appreciation are positive.

In particular, program characteristics were identified that *facilitated* the implementation process, demonstrating the impact of the implementation studies conducted on earlier versions of Krachtvoer [27, 28]. These included the active learning methods and digital program components in simple language and attractive looks that were reported as appropriate for prevocational students.

A key finding of the current study was that “minor” program characteristics, such as the possibility to color small drawings on the lesson materials, could increase responsiveness. However, this also proved to be the case in the inverse. Appreciation and implementation also became less optimal by “minor” program aspects, related to not only content, but also layout and technology, that mismatched contextual factors. Most mismatches could be quickly resolved due to the continuous micro‐process cycles in our action‐oriented research [34].

In and between unique school contexts, differences were found between teachers in didactical skills, prior knowledge of nutrition, perceptions about the use of varied learning methods, and digital technology skills, and this influenced implementation. Relevant differences between students were also found in terms of maturity, attention span, tech‐savviness, creativity, and peer influence. The differences found in and between unique school contexts were quite large, despite the fact that it already concerned the specific context of prevocational schools and students aged 12–14.

All teachers adapted the program by choosing from the alternative working methods offered, but also by making other adjustments to lesson components. Teachers with more years of teaching experience drew on their experience here. Teachers felt encouraged and helped to adapt to the program by the guidance on the website and they proved resourceful enough to adapt program components in situations where teaching was disrupted while adhering to the program goal. Most adjustments were desirable from the perspective of the program's developers. However, some less experienced and digitally skilled teachers conducted adaptations that interfered with implementation as intended by the developers. The freedom of teachers to balance implementation decisions, also described as adaptive management [35], placed a burden on some teachers and might indicate a potential capacity building need in balancing implementation decisions [36], as this resulted in undesirable skipping or shortening of program components.

Because of the differences in and between unique school contexts, the program did not yet offer sufficient adaptation options, including offline alternatives to digital components, because these were not suitable for all students and in all schools. This need has become even more topical by the recently updated Dutch government policy on the use of mobile phones by students in the classroom.

### 4.2 Implications for School‐Based HP

The development of HP programs that are sensitive to and can be adapted to different implementation contexts, co‐creation from the start of program development, and the development of strategies to support implementation are among the implications for HP in schools that could already be deduced from previous scientific work that formed the starting point of the current study. Based on the present study, their importance has become more evident, and some implications can be further refined. Since many contextual differences exist between teachers, students, and schools, and even small contextual mismatches affect implementation, school‐based HP programs must be adapted to the general implementation context (e.g., prevocational schools), but users must also be able to further adapt it to their own unique context. This means offering opportunities to adapt the program to differences in educational level, existing social norms and group behavior, attention span, tech‐savviness, and age of students. HP programs and user support programs must also address teacher differences in adaptive management technological skills, as well as motivation. By developing pre‐structured materials, offering sufficient options for adaptation, and capacity building among teachers in balancing implementation decisions, it must be ensured that the core of program activities is not lost during the adaptation process. All of the above implies that sustaining HP programs that fit the context requires continuous and co‐creating efforts from all stakeholders, as the general as well as the unique contexts are constantly changing.

### 4.3 Limitations

The study was conducted during a short research period in a small number of schools, which limits the generalizability of the findings. However, within the current research context, the study provided many useful insights, which we attribute to the combination of data collection methods and the comprehensive conceptual framework of the study, including a comprehensive perspective on different implementation fidelity indicators [37]. Another limitation of the current study was that the support (and capacity building) program could not be examined because teachers hardly used it in the context of the study.

## 5 Conclusions

The action‐oriented research with micro‐process cycles involving teachers, students, and program developers in the careful development and trial of program aspects proved appropriate for identifying contextual differences between teachers, students, and schools, and for quickly identifying and eliminating contextual mismatches and technological glitches of the program. Thus, the study's aim to strengthen the Krachtvoer program has been achieved. In addition, general lessons have been learned that call for ongoing and concerted efforts by all stakeholders to ensure that HP programs continue to fit the ever‐changing general implementation context and to provide sufficient alternative options and support for users to adapt programs to their unique context. Finally, more research is needed on the need for any form of capacity building among program implementers in balancing program fidelity and adaptation and on the potential of implementation support tailored to the implementer.

## Ethics Statement

In line with Dutch law and policies of the research institute regarding this type of non‐medical research with human subjects at the time of the study, no ethical clearance was required from an ethics review committee.

## Consent

Written informed consent was obtained from schools and teachers prior to participation in the action‐oriented research.

## Conflicts of Interest

The authors declare no conflicts of interest.
